# Overexpression of KLF5 is associated with poor survival and G1/S progression in pancreatic cancer

**DOI:** 10.18632/aging.102096

**Published:** 2019-07-21

**Authors:** Yilong Li, Rui Kong, Hongze Chen, Zhongjie Zhao, Le Li, Jiating Li, Jisheng Hu, Guangquan Zhang, Shangha Pan, Yongwei Wang, Gang Wang, Hua Chen, Bei Sun

**Affiliations:** 1 Department of Pancreatic and Biliary Surgery, The First Affiliated Hospital of Harbin Medical University, Harbin 150001, China; 2 Key Laboratory of Hepatosplenic Surgery, Ministry of Education, Harbin 150001, China

**Keywords:** pancreatic cancer, KLF5, microRNA, long-term survival, short-term survival

## Abstract

Despite improvements in surgical procedures and comprehensive therapies, pancreatic cancer remains one of the most aggressive and deadly human malignancies. It is therefore necessary to determine which cellular mediators associate with prognosis in pancreatic cancer so as to improve the treatment of this disease. In the present study, mRNA array and immunohistochemical analyses showed that KLF5 is highly expressed in tissue samples from three short-surviving patients with pancreatic cancer. Survival analysis using data from The Cancer Genome Atlas showed that patients highly expressing KLF5 exhibited shorter overall and tumor-free survival times. Mechanistically, KLF5 promoted expression of E2F1, cyclin D1 and Rad51, while inhibiting expression of p16 in pancreatic cancer cells. Finally, flow cytometric analyses verified that KLF5 promotes G1/S progression of the cell cycle in pancreatic cancer cells. Collectively, these findings demonstrate that KLF5 is an important prognostic biomarker in pancreatic cancer patients, and they shed light on the molecular mechanism by which KLF5 stimulates cell cycle progression in pancreatic cancer.

## INTRODUCTION

Pancreatic cancer is one of the most aggressive and deadly human malignancies, with a 5-year survival rate of only 8% [1]. The American Cancer Society estimates that in 2018, new pancreatic cancer cases will be the fourth leading cause of death among all cancer types in both males and females [1]. This unfavorable prognosis is primarily due the aggressive biological behavior of pancreatic tumors, which show early metastasis, poor responses to chemotherapy and radiotherapy, and late diagnosis [2]. Many recent studies have focused on the genetic and epigenetic alterations that inactivate tumor suppressor genes and activate oncogenes in pancreatic cancer [3–5]. However, the biological processes responsible for the poor prognosis in pancreatic cancer are still not totally clear.

Krüppel-like factor 5 (KLF5) is a member of Krüppel-like factor (KLF) family and is widely expressed in different tissues. KLF5 binds to GC-rich DNA sequences via its zinc finger domain [6, 7]. As a transcription factor, KLF5 regulates a number of important target genes, including PDGF-α [8], cyclin D1 [9, 10], survivin [11] and p21 [12]. Within cancer cells, KLF5 has an important impact on cell survival, cell cycling, apoptosis and migration [7, 13]. In breast cancer, for example, KLF5 promotes cell proliferation, migration and invasion by up-regulating expression of TNFAIP2 [14]. Similarly, KLF5 has also been shown to promote breast cancer cell proliferation and survival by upregulating expression of FGF-BP and mPGES1 [15, 16]. In pancreatic cancer, a recent study showed that KLF5 promotes cell proliferation, acinar-to-ductal metaplasia, pancreatic intraepithelial neoplasia, and tumor growth [17].

KLF5’s key role is also reflected in its regulation of microRNA. MicroRNAs are a family of non-coding RNAs, approximately 21–23 nucleotides in length, which play significant roles in a variety of cellular processes [18, 19]. MicroRNAs negatively regulate gene expression posttranscriptionally by targeting the 3’-untranslated regions (3’-UTR) of target mRNAs through complementary base pairing, thereby inhibiting their translation [20]. MicroRNAs reportedly target more than 30% of human genes [21], greatly impacting cell survival, apoptosis, proliferation, invasion and metastasis, as well as angiogenesis and resistance to chemotherapy, all of which can affect the initiation and progression of human cancers [22–24].

Earlier studies revealed that KLF5 directly induces the transcription of the miR-200 family of microRNAs by binding to the GC boxes in their promoters [25]. In the present study, we used data from The Cancer Genome Atlas (TCGA) and an mRNA array to confirm that KLF5 expression is a key determinant of survival time in pancreatic cancer patients after surgery as well as a prognostic biomarker in pancreatic cancer. Moreover, we used bioinformatics analysis investigate the target genes whose transcription is affected by KLF5 in pancreatic cancer. Our findings indicate that KLF5 is a critical oncogene in human pancreatic cancer.

## RESULTS

### KLF5 is differentially expressed between short- and long-surviving pancreatic cancer patients

**Figure 1:**
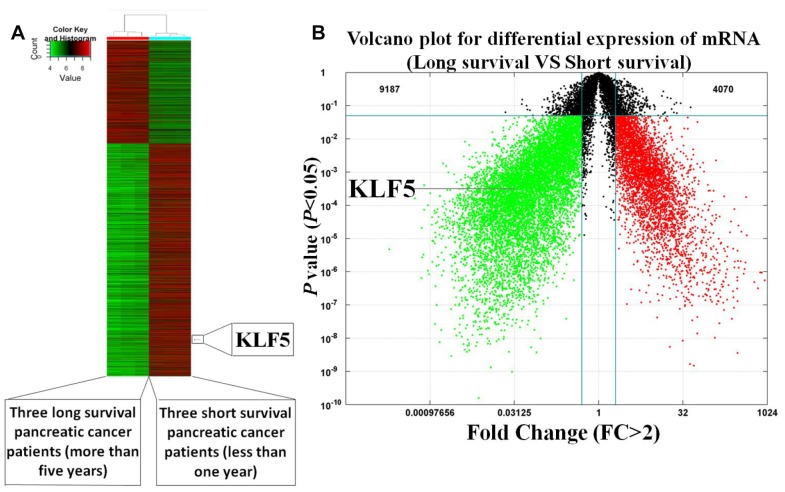
**Heat map and volcano plot illustrating the gene expression profiles of short- and long-surviving pancreatic cancer patients.** (**A**) Heat map of illustrating the gene expression profiles. Strongly expressed genes are shown in red, while weakly expressed genes are shone in green. KLF5 expression is higher in three short-surviving patients than in three long-surviving patients. (**B**) Volcano plot showing differential gene expression (*P*<0.05, FC>2).

**Figure 2:**
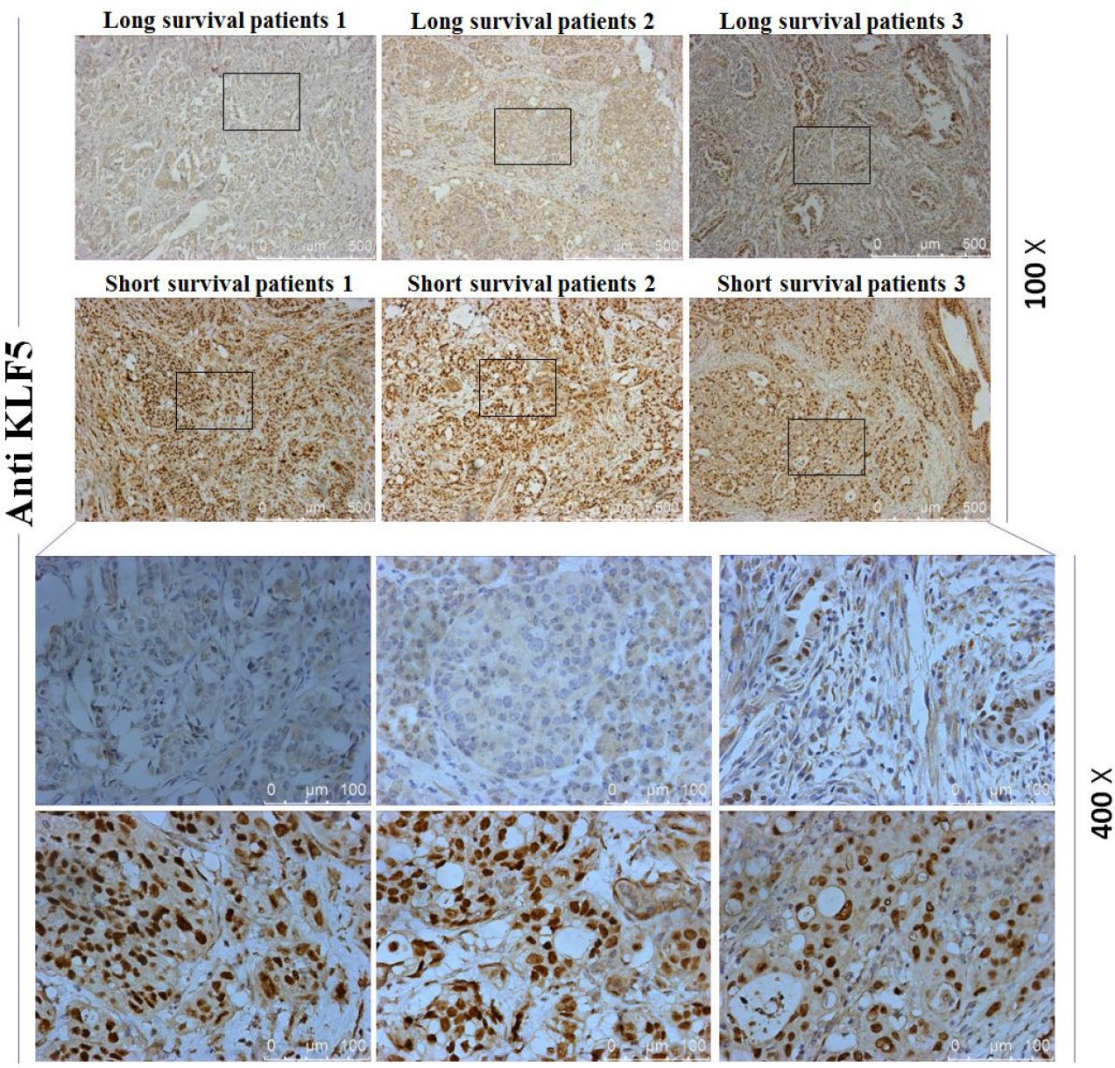
**Immunohistochemical staining for KLF5.** Staining for KLF5 is stronger in three short-surviving patients than in three long-surviving patients.

To investigate whether the high or low KLF5 expression is a molecular event affecting survival of pancreatic cancer patients, mRNA array and immunohistochemical analyses were used to compare levels of KLF5 expression between tissues samples from three long-surviving and three short-surviving pancreatic cancer patients. The postsurgical survival times of the long-surviving patients were more than 5 years, while those of the short-surviving patients were less than 1 year. The heat map summarizing the mRNA array data shown in Figure 1A illustrates the significantly different expression profiles between the two patient groups. KLF5 was more highly expressed in tissues from the short-surviving patients than in those from the long-surviving patients. Moreover, the volcano plots in Figure 1B show that KLF5 expression is nearly 31 times higher in the tissue from short-surviving than long-surviving patients (*P*=0.0003). In addition, immunohistochemical staining for KLF5 protein was stronger in tissues from short-surviving than long-surviving patients (Figure 2), which is consistent with the mRNA array data.

### The KLF5 expression level is prognostic for overall survival in pancreatic cancer patients

**Figure 3:**
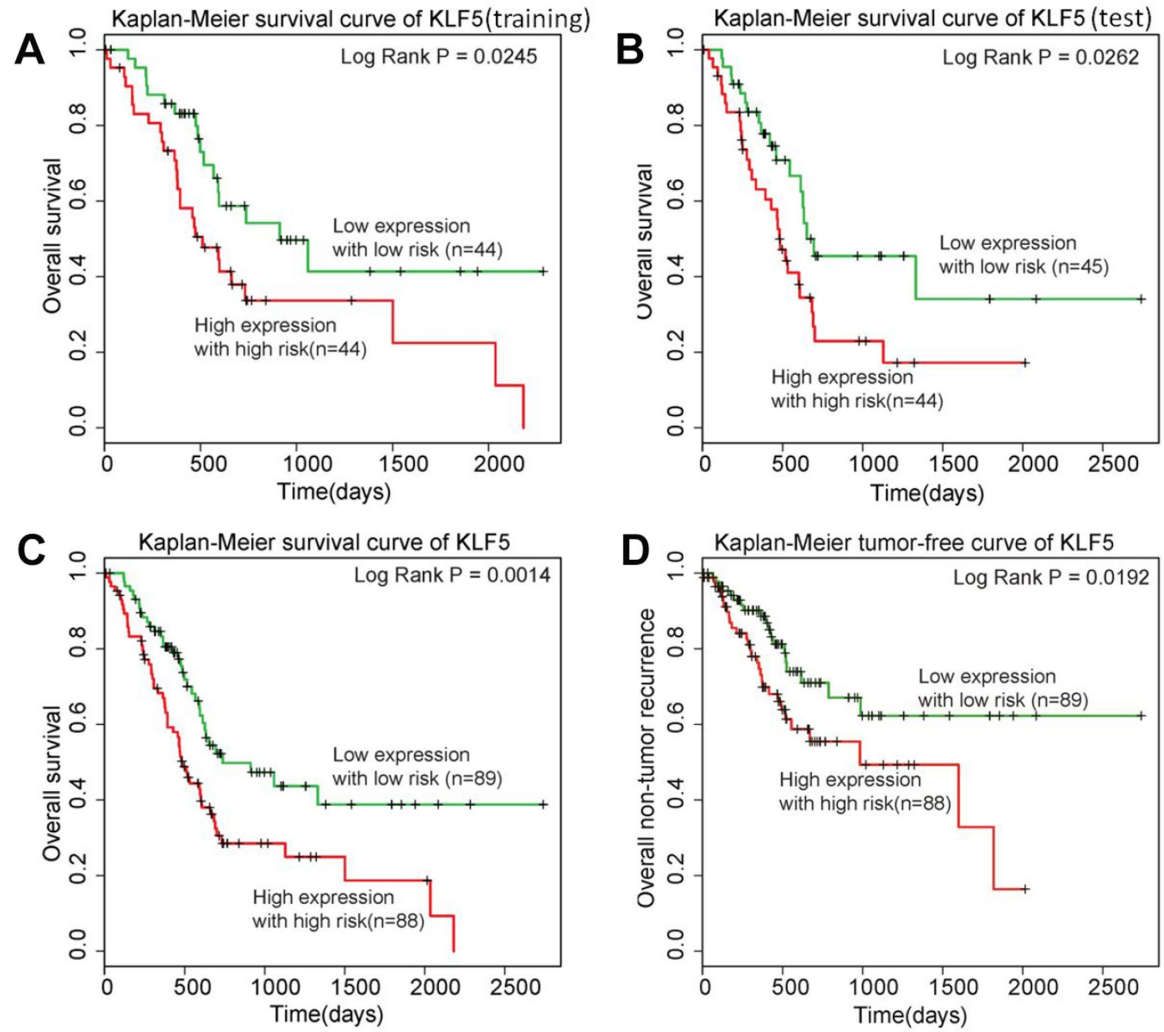
**KLF5 is highly associated with pancreatic cancer patient prognosis.** (**A**) Patients in the training group with high expression of KLF5 have shorter survival times than patients with low KLF5 expression. (**B**) Patients in the test group with high expression of KLF5 have shorter survival times than patients with KLF5 low expression. (**C**) Among all 177 patients included, those with high KLF5 expression have shorter survival times than those with low KLF5 expression. (**D**) Patients exhibiting high KLF5 expression have shorter tumor-free survival than those exhibiting low expression of KLF5.

To determine whether KLF5 could be used as a prognostic biomarker in patients with pancreatic cancer, we built the risk score model described in the Methods section. Briefly, using a dataset for 177 pancreatic cancer patients from TGCA, we randomly assigned patients to a training group (n = 88) or a test group (n = 89). Then using survival analysis, we divided patients in the training group into better and poorer survival subgroups and determined whether there was a significant difference in KLF5 expression between the two subgroups (Figure 3A). We found that patients exhibiting high KLF5 expression had shorter survival times than those exhibiting low KLF5 expression. We then confirmed that KLF5 expression could be used to divide patients in the test group into better and poorer survival subgroups using the same cut-off point as in the training group (Figure 3B). In the test group, patients showing low expression of KLF5 had longer survival times than those showing high expression of KLF5. Finally, KLF5 expression was used to divide all 177 patients into better and poorer survival groups (Figure 3C). These results indicate that the KLF5 expression level is indicative of the postsurgical survival time in pancreatic cancer patients. The KLF5 expression level thus appears to be a prognotic biomarker in patients with pancreatic cancer.

### KLF5 is highly associated with tumor-free survival time in pancreatic cancer patients

Owing to its aggressiveness, pancreatic cancer patients who experience tumor recurrence generally die relatively soon thereafter. In other words, analysis of overall survival does not totally reflect KLF5’s key role in determining the prognosis of pancreatic cancer patients. Therefore, a similar analysis was carried out for tumor-free survival using tumor event data from TCGA. Notably, the 177 patients in the TCGA dataset could be clearly better and poorer tumor-free survival groups based on the KLF5 expression level (Figure 3D). As with overall survival, patients exhibiting low KLF5 expression had significantly better tumor-free survival times after surgery than those exhibiting high KLF5 expression.

### Regulatory genes targeted by KLF5 in pancreatic cancer

To investigate the mechanism by which high KLF5 expression leads to a poorer prognosis in pancreatic cancer patients, we examined the important genes activated or inhibited by KLF5 in the pancreatic cancer pathway extracted in the KEGG. The important genes activated or inhibited by KLF5 are listed in the Supplementary Material [26]. In that study, KLF5 was knocked out in the CFPAC-1 pancreatic cancer cell line using CRISPR/Cas9-mediated genome editing. We identified genes differentially expressed between the KLF5-KO and control clones based on two criteria: 1) because KLF5 is a transcription factor, the selected differentially expressed genes were downregulated in KLF5-KO cells; 2) differentially expressed genes were components of the pancreatic cancer pathway extracted in KEGG.

**Figure 4:**
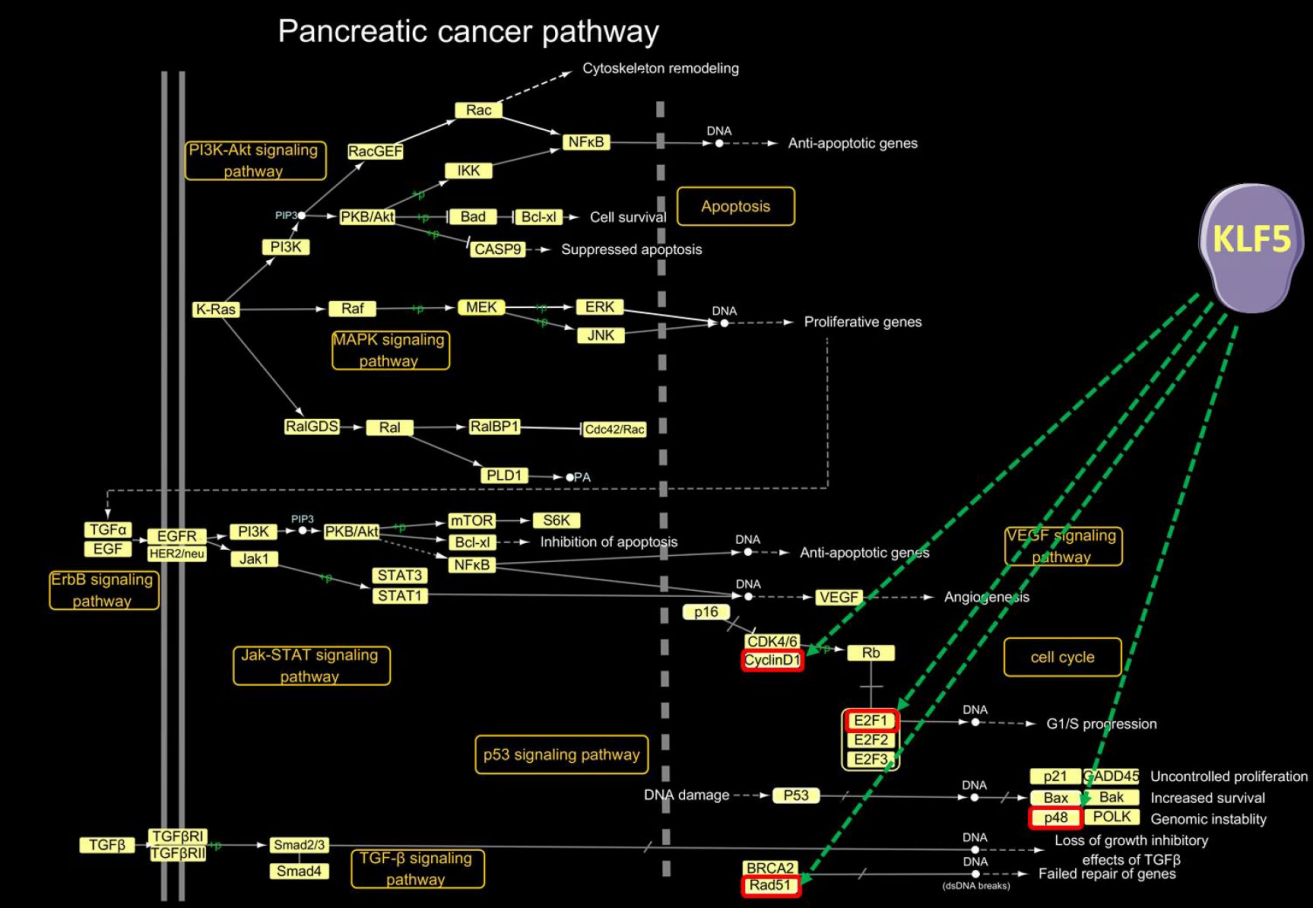
**KLF5-targeted genes in the pancreatic cancer pathway.** In the pancreatic cancer pathway extracted from the KEGG PATHWAY database (**http://www.genome.jp/kegg/pathway.html**), cyclin D1, E2F1, p48 and Rad51 were activated by KLF5.

The pancreatic cancer pathway extracted in KEGG is a comprehensive pathway that includes the PI3K-Akt, MAPK, ErbB, Jak-STAT, VEGF, p53, and TGF-β signaling pathways, as well as the cell cycle and apoptosis pathways. A detailed examination of KLF5’s target genes within the pancreatic pathway map is shown in Figure 4. KLF5-activated genes include cyclin D1, E2F1, p48 and Rad51. Upregulation of these genes led to G1/S progression, uncontrolled proliferation, increased survival, genomic instability and failed repair of genes.

### Regulatory microRNAs targeted by KLF5 in pancreatic cancer

To further examine the mechanisms by which KLF5 expression leads to a poorer prognosis in pancreatic cancer patients, we identified the microRNAs activated by KLF5 and their target genes and evaluated their roles in the pancreatic cancer pathway extracted in KEGG. The important microRNAs regulated by KLF5 are listed in the Supplementary Material for reference 25. In that study, KLF5 was knocked down using lentiviruses encoding shRNAs targeting KLF5 mRNA, and all microRNAs were detected using a realtime-qPCR-based microRNA array. We identified differentially expressed microRNAs based on three criteria: 1) because KLF5 is a transcription factor, the selected differentially expressed microRNAs were downregulated in KLF5-knockdown cells; 2) differentially expressed microRNAs inhibited expression of tumor suppressors in the pancreatic cancer pathway extracted in KEGG; 3) interactions between selected differentially expressed microRNAs and target genes such as tumor suppressors and components of the pancreatic cancer pathway were experimentally verified using the miRTarBase (24304892) database.

**Figure 5:**
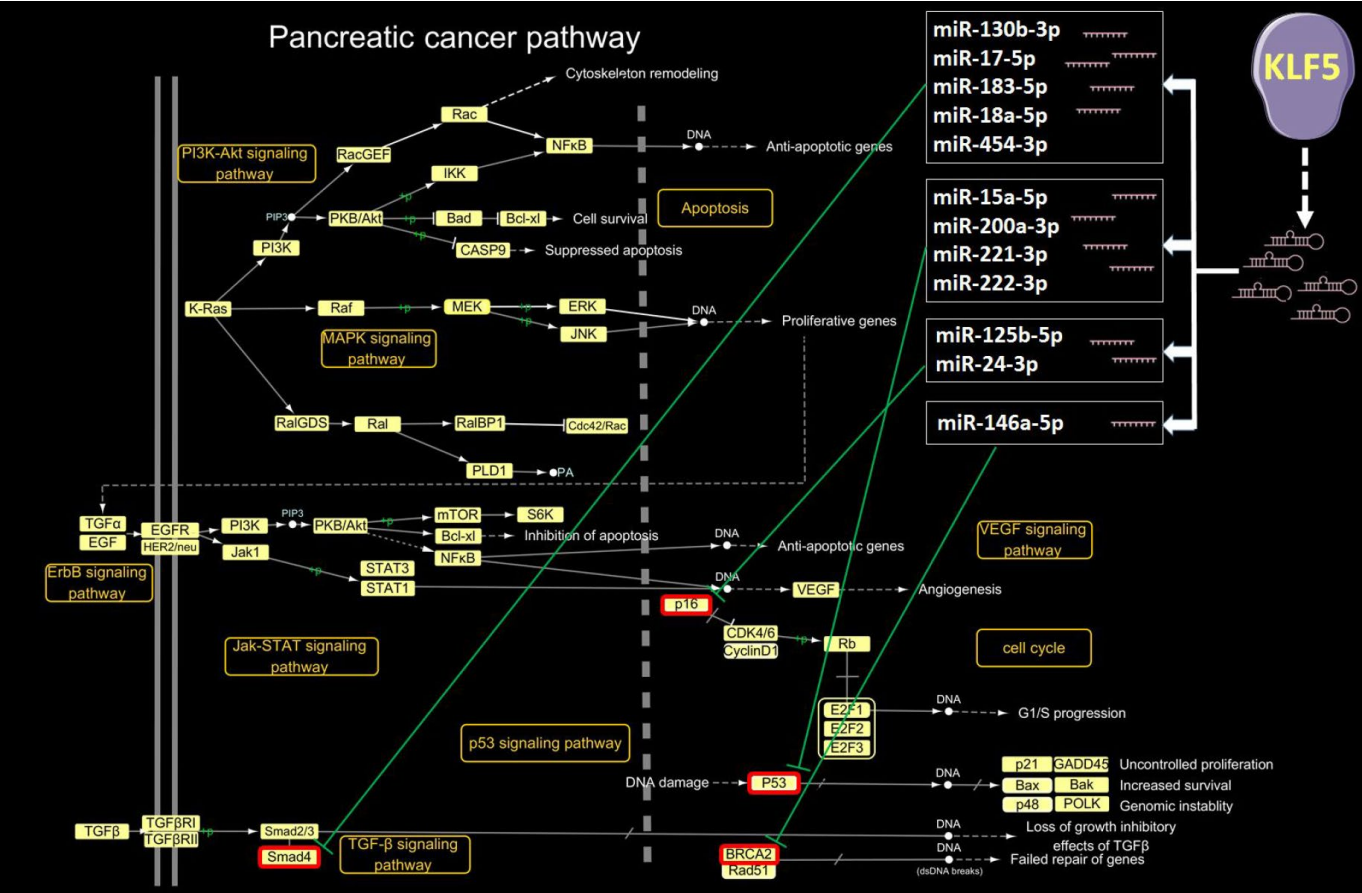
**KLF5-targeted microRNAs in the pancreatic cancer pathway.** In the pancreatic cancer pathway extracted from the KEGG PATHWAY database (**http://www.genome.jp/kegg/pathway.html**), 12 microRNAs activated by KLF5 inhibit Smad4, p53, p16 and BRCA2.

Our detailed examination of microRNAs activated by KLF5 and their target genes within the pancreatic pathway are shown in Figure 5. MicroRNAs activated by KLF5 include miR-130b-3p, miR-15a-5p, miR-17-5p, miR-183-5p, miR-18a-5p, miR-200a-3p, miR-221-3p, miR-222-3p, miR-454-3p, miR-125b-5p, miR-146a-5p and miR-24-3p. Smad4 is downregulated by miR-130b-3p, miR-17-5p, miR-183-5p, miR-18a-5p and miR-454-3p; p53 is downregulated by miR-15a-5p, miR-200a-3p, miR-221-3p and miR-222-3p; p16 is down-regulated by miR-125b-5p and miR-24-3p; and BRCA2 is down-regulated by miR-146a-5p. A microRNA microarray was also used to assess expression of the aforementioned KLF5-activated microRNAs in tissues from three long-surviving and three short-surviving pancreatic cancer patients. As shown in (Supplementary Figure 1), miR-183-5p, miR-222-3p, miR-221-3p, miR-200a-3p, miR-125-5p and miR-24-3p were all highly expressed in tissue samples from short-surviving patients. However, the *P* values for only miR-200a-3p and miR-183-5p were less than 0.05.

### GO and KEGG enrichment analysis of regulatory genes targeted by KLF5

**Figure 6:**
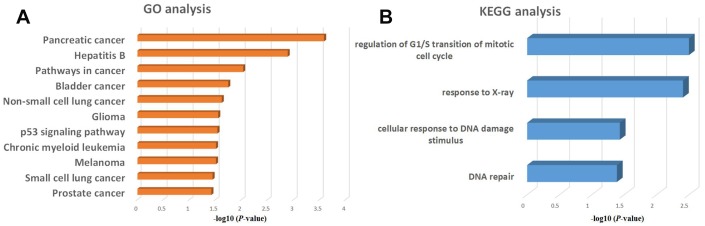
**GO and KEGG enrichment analysis of regulatory genes potentially targeted by KLF5.** (**A**) GO analysis of KLF5-targeted genes. (**B**) KEGG analysis of KLF5-targeted genes.

To gain additional insight into the functional roles of genes activated by KLF5, we performed GO and KEGG functional enrichment analyses for cyclin D1, E2F1, p48 and Rad51. GO analysis revealed that these four genes were associated with a variety of cancers, especially pancreatic cancer (Figure 6A). In a KEGG analysis, these four genes were associated with regulation of G1/S transition of the mitotic cell cycle, response to X-ray, cellular response to DNA damage, and DNA repair (Figure 6B).

### GO and KEGG enrichment analysis of regulatory microRNAs targeted by KLF5

**Figure 7:**
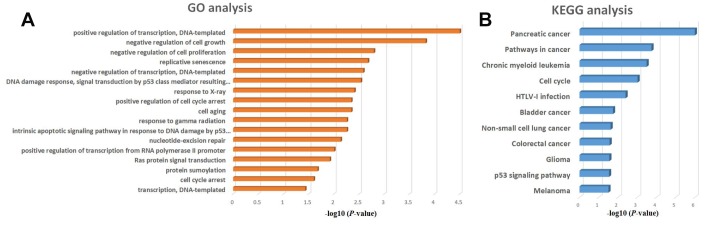
**GO and KEGG enrichment analysis of regulatory microRNAs potentially targeted by KLF5.** (**A**) GO analysis of KLF5-targeted microRNAs of KLF5. (**B**) KEGG analysis of KLF5-targeted microRNAs.

To obtain additional insight into the functional roles of the microRNAs activated by KLF5, we performed GO and KEGG functional enrichment analyses for miR-130b-3p, miR-15a-5p, miR-17-5p, miR-183-5p, miR-18a-5p, miR-200a-3p, miR-221-3p, miR-222-3p, miR-454-3p, miR-125b-5p, miR-146a-5p and miR-24-3p. The GO analysis showed that these twelve microRNAs are significantly associated with positive regulation of transcription (Figure 7A), while the KEGG analysis showed that they are associated a variety of cancers, especially pancreatic cancer (Figure 7B).

### Confirmation of regulatory relationships between KLF5 and its target genes

**Figure 8:**
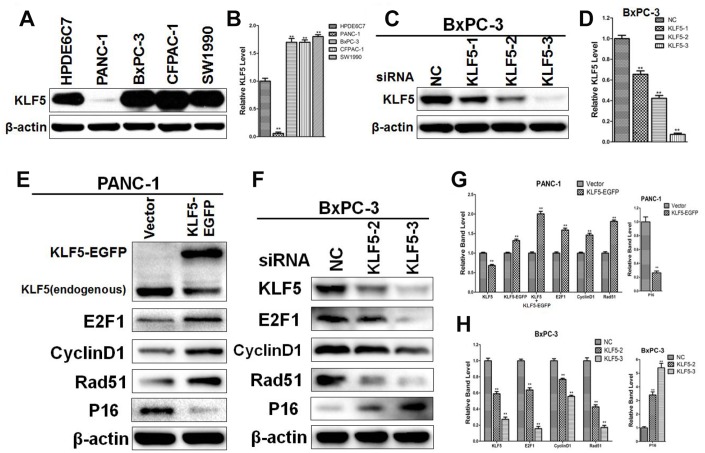
**Western blot analysis confirming the regulatory relationships between KLF5 and its target genes** (**A**) KLF5 expression in pancreatic cancer cell lines and a normal pancreatic cell line. (**B**) The density of each band was measured and normalized to the β-actin band. * *P* <0.05, ** *P* <0.01 vs. the HPDE6C7 group. (**C**) KLF5 expression after transfection of cell with KLF5 siRNA. (**D**) The density of each band was measured and normalized to the β-actin band. * *P* <0.05, ** *P* <0.01 vs. the negative control (NC) group. (**E**) PANC-1 cells were transfected for 48 h with empty vector or KLF5-EGFP plasmid, after which the protein extracts were assayed by western blotting. β-actin was used as a protein loading control. **(F)** BxPC-3 cells were transfected for 48 h with negative control siRNA, siKLF5-2 or siKLF5-3, after which the protein extracts were assayed by western blotting. β-actin was used as a protein loading control. (**G**) The density of each band was measured and normalized to the β-actin band. * *P* <0.05, ** *P* <0.01 vs. the empty vector group. (**H**) The density of each band was measured and normalized to the β-actin band. * *P* <0.05, ** *P* <0.01 vs. the NC group.

To determine the regulatory relationships between KLF5 and its target genes, we initially compared the levels of KLF5 expression between four pancreatic cancer cell lines (PANC-1, BxPC-3, CFPAC-1, and SW1990) and a line of normal pancreatic cells (HPDE6C7). As shown in Figure 8A and 8B, KLF5 expression was higher in BxPC-3, CFPAC-1 and SW1990 cells and was lower in PANC-1 cells than in HPDE6C7 cells. We then used western blotting to determine that siKLF5-2 and siKLF5-3 are the most effective siRNAs for knocking down endogenous KLF5 expression in BxPC-3 cells (Figure 8C and 8D). Similar results were also obtained in CFPAC-1 cells (Supplementary Figure 2).

To investigate the regulatory relationships between KLF5 and its target genes identified from the Supplementary Material of reference 26 and through systematic analysis, we transfected PANC-1 and BxPC-3 cells with KLF5-EGFP plasmid or siRNA targeting KLF5. We then used western blotting to assess expression of KLF5 protein and its target genes, including E2F1, cyclin D1, Rad51, p48 and p16. As shown in Figure 8E, transfection of KLF5-EGFP led to KLF5 overexpression, which in turn increased E2F1, cyclin D1 and Rad51 levels and reduced p16 levels in PANC-1 cells. Conversely, knocking down endogenous KLF5 using siRNA reduced levels of E2F1, cyclin D1 and Rad51 and increased levels of p16 in BxPC-3 cells (Figure 8F). Similar results were also obtained in CFPAC-1 cells (Supplementary Figure 2). We did not detect a regulatory relationship between KLF5 and p48 in PANC-1, BxPC-3 or CFPAC-1 cells. KLF5 overexpression also increased cyclin B1 and CDC2 levels in PANC-1 cells, while endogenous KLF5 knockdown reduced cyclin B1 and CDC2 levels in BxPC-3 and CFPAC-1 cells (Supplementary Figure 3). Immunohistochemical analysis revealed that staining for cyclin D1, E2F1 and Rad51, which were all increased by KLF5, was stronger in tissues from short-surviving than long-surviving patients. By contrast, staining for p16, which was decreased by KLF5, was stronger in tissues from long-surviving than short-surviving patients (Supplementary Figures 4–7).

### KLF5 promotes G1/S progression in pancreatic cancer cells

**Figure 9:**
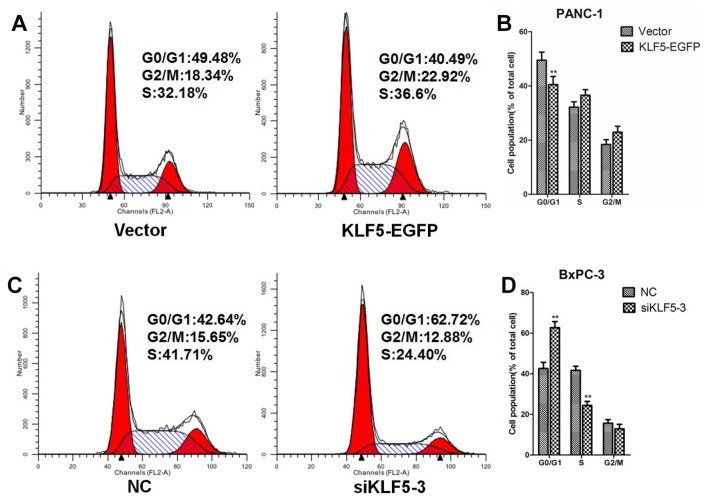
**KLF5 promotes G1/S progression in pancreatic cancer cells.** (**A**) PANC-1 cells were transfected for 48 h with empty vector or KLF5-EGFP plasmid, after which the cells were collected for flow cytometric analysis. (**B**) * *P* <0.05, ** *P* <0.01 vs. the empty vector group. (**C**) BxPC-3 cells were transfected for 48 h with NC siRNA or siKLF5-3, after which the cells were collected for flow cytometric analysis. (**D**) * *P* <0.05, ** *P* <0.01 vs. the NC group.

To investigate whether KLF5 expression level affects the cell cycle, we performed a flow cytometric analysis of the cellular DNA content. We found that overexpressing KLF5-EGFP promoted G1/S progression in PANC-1 cells (Figure 9A and 9B). Conversely, knocking down endogenous KLF5 expression using siKLF5-3 led to cell cycle arrest at G1 phase in BxPC-3 cells (Figure 9C and 9D). Similar results were also obtained using CCK8 assays (Supplementary Figure 8). KLF5-EGFP overexpression promoted PANC-1 cell proliferation, while KLF5 knockdown inhibited BxPC-3 cell proliferation.

## DISCUSSION

Pancreatic cancer is associated with a poor prognosis and a low 5-year survival rate. Despite great effort to improve treatments, the American Cancer Society estimates that 44,330 Americans died from pancreatic cancer in 2018, and that the 5-year survival rate is only 8% [1]. The main reasons for the low survival rate are related to 1) the pancreas being situated deep within the anatomical structure of the human body and patients being asymptomatic until the disease has reached an advanced stage [2]; 2) only about 20% of pancreatic cancer patients are candidates for surgical resection [27]; and 3) the malignant behavior of pancreatic cancer often leads to early metastasis, early recurrence, and resistance to chemotherapy and radiotherapy [2]. In that regard, previous studies showed that KLF5 promotes breast cancer cell migration and invasion by upregulating transcription of TNFAIP2 [28], and that KLF5 controls keratinocyte migration via the integrin-linked kinase [29]. These studies suggest that KLF5 may be a key mediator of early metastasis of pancreatic cancer.

To our knowledge, this is the first study to examine differences in the gene expression profile between tissue samples from long- and short-surviving pancreatic cancer patients. After determining the differentially expressed genes, we found that KLF5 is highly expressed in short-surviving patients. Moreover, using a TCGA dataset from 177 pancreatic cancer patients, we confirmed that higher/lower KLF5 expression is predictive of poorer/better overall and tumor-free survival, and that KLF5 is the key determinant of survival time in pancreatic cancer patients. The mechanism by which KLF5 affects survival time appears related to its actions as a transcription factor promoting oncogene expression as well as expression of microRNAs that downregulate expression of tumor suppressors. Notably, signaling in multiple pathways contributes to the regulation of KLF5 expression at the transcriptional and posttranslational levels [30]. For example, KLF5 is reportedly induced via the Ras-MAPK signaling pathway [31] and is also a Wnt-responsive gene [32]. However, the precise reasons why high KLF5 expression shortens the survival of pancreatic cancer patients remains unclear.

Cyclin D1 forms active complexes with CDK4 or CDK6. These complexes can phosphorylate retinoblastoma protein, leading to G1/S progression in the cell cycle [33]. This function of cyclin D1 suggests its potential involvement in the regulation of cell cycle progression in pancreatic cancer. Factors controlling cyclin D1 transcription include β-catenin, EGFR, PI3K and NF-κB [33]. In addition, upon induction by angiotensin II, KLF5 binds to the promotor region of cyclin D1 gene in vascular smooth muscle cells, thereby enhancing its expression [34]. In the present study, KLF5 promoted expression of cyclin D1, which accelerated cell cycling. Taken together, these studies suggest KLF5 may promote cyclin D1 gene expression directly by binding to its promoter in pancreatic cancer cells. This experimental result may partially explain the adverse effect of KLF5 on survival time in pancreatic cancer patients.

The E2F family of transcription factors are critical regulators of cell cycling and apoptosis. Some E2Fs (e.g., E2F1 and E2F3) act as activators, while others (e.g., E2F4 and E2F5) act as repressors [35]. E2F1 is composed of 437 amino acids and is often seen during late G1/S phase [35]. E2F1 is involved in such cellular processes as migration, differentiation, metabolism, cell reprogramming, proliferation and cell cycling [36–38]. In hepatocellular carcinoma cells, E2F1 promotes cell proliferation and suppresses apoptosis by inducing c-Myc expression and upregulating SKP2 expression [39]. During the cell cycle, E2F1 is controlled by the retinoblastoma protein Rb [35]. When not in its hyperphosphorylated form, Rb complexes with E2F1and inhibits its DNA binding [35]. However, when Rb is phosphorylated by CDKs, E2F1 is released from the Rb/E2F1 complex, after which it promotes transcription of its target genes, which include CDC2, CDC25A, cyclin D1 and cyclin E [35]. In the present study, we observed that KLF5 promotes E2F1 expression, which in turn accelerated cell cycling. However, whether KLF5 acts as a transcription factor, binding to the of E2F1 promoter, remains unclear. Nonetheless, our results may explain in part how KLF5 affects survival time in pancreatic cancer patients.

Rad51 is a multifunctional protein involved in DNA replication and homologous recombination repair and plays a significant role in cancer development [40]. It reportedly promotes cell survival, prevents accumulation of replication-associated double-strand breaks, and maintains genome stability [41]. There is no conclusive evidence of a relationship between KLF5 and Rad51. However, our findings indicate that KLF5 can promote the expression of Rad51. Whether KLF5 binds to the Rad51 promoter to stimulate its gene expression and whether Rad51 can enhance the ability of pancreatic cancer cells to resist survival pressure from chemotherapeutic agents such as gemcitabine remain to be determined.

In the pancreatic cancer KEGG pathway map, the functions of p16, p53, Smad4 and BRCA2 are as tumor suppressors. p16 is well known to bind CDK4/6, inhibit the cyclin D-CDK4/6 complex, negatively regulate cellular proliferation, and positively inhibit tumor formation [42]. To treat cancer, many strategies have focused on blocking the p16-CDK4-cyclinD-Rb axis pharmacologically [43]. Interestingly, our experimental results indicate that KLF5 downregulates p16 expression, perhaps through induction of miR-125b-5p and miR-24-3p. The microRNA microarray also showed that, as with KLF5, miR-125-5p and miR-24-3p were highly expressed in tissue samples from short-surviving patients. This finding may also contribute to the explanation of why pancreatic cancer patients showing high KLF5 expression exhibit shorter survival times. That said, it remains to be shown whether KLF5 binds to the miR-125-5p or miR-24-3p promoter to stimulate their expression.

In summary, we found that levels of KLF5 are higher in tissue samples from short-surviving pancreatic cancer patients than in samples from long-surviving patients. Moreover, both overall and tumor-free survival were shorter in patients exhibiting high KLF5 expression. From a mechanistic perspective, we observed that KLF5 promotes expression of cyclin D1, E2F1 and Rad51, which could lead to G1/S progression, uncontrolled cell proliferation, increased cell survival and drug resistance. In addition, KLF5 inhibits expression of the tumor suppressor p16 via microRNAs, which would likely further increase the malignancy of pancreatic cancer cells. These characteristics may make KLF5 a useful therapeutic target in pancreatic cancer.

## MATERIALS AND METHODS

### Patients and specimens

This study was approved by the Ethics Committee of the First Affiliated Hospital of Harbin Medical University (201861). The six PDAC specimens used in this experiment were obtained from patients who underwent pancreaticoduodenectomy or distal pancreatectomy for pancreatic head cancer or pancreatic body-tail cancer in the Department of Pancreatic and Biliary Surgery between January 2006 and January 2018. The patients’ detailed clinicopathologic characteristics are listed in Table 1.

**Table 1:** The clinical characteristics of 6 pancreatic cancer patients (3 Long survival VS 3 Short survival).

	**Long survival patients 1**	**Long survival patients 2**	**Long survival patients 3**	**Short survival patients 1**	**Short survival patients 2**	**Short survival patients 3**
Age (years)	83	50	65	53	49	66
Gender	Male	Female	Male	Male	Female	Male
Vital status	Alive	Dead	Dead	Dead	Dead	Dead
Histologic diagnosis	PDAC	PDAC	PDAC	PDAC	PDAC	PDAC
Tumor grade	Moderately differentiated	Moderately differentiated	Well differentiated	Poorly differentiated	Moderately and poorly differentiated	Moderately and poorly differentiated
Site of disease	Pancreatic body and tail	Pancreatic body and tail	Pancreatic head	Pancreatic head	Pancreatic body and tail	Pancreatic body and tail
Surgery date	29th March 2012	3th January 2007	26th September 2008	14th December 2015	14th April 2016	18th February 2016
Death date	Alive	November 2014	October 2013	August 2016	August 2016	October 2016
Survival time after surgery	More than 5 years	More than 5 years	More than 5 years	Less than 1 year	Less than 1 year	Less than 1 year

### RNA extraction

The method of RNA extraction was described previously [20]. Briefly, total RNA was extracted and isolated using TRIzol reagent (Invitrogen Life Technologies) according to the manufacturer’s instructions. RNA quantity and quality were measured using a NanoDrop ND-1000 spectrophotometer. Acceptable OD A260/A280 ratios were between 1.8 and 2.1 (the target is approximately 2.0 for pure RNA), and OD A260/A230 ratios were greater than 1.8. RNA integrity was assessed using standard denaturing agarose gel electrophoresis.

### Protein-coding transcript microarray procedure

Microarray and data analysis were performed by KangChen Biotechnology, Shanghai, China. The Arraystar Human LncRNA Microarray V4.0 is designed for global profiling of human LncRNAs and protein-coding transcripts. Around 40,173 LncRNAs and 20,730 coding transcripts can be detected by this third-generation LncRNA microarray. Sample labeling and array hybridization were performed using the Agilent One-Color Microarray-Based Gene Expression Analysis protocol (Agilent Technology) with minor modifications. Briefly, mRNA was purified from total RNA after removal of rRNA (mRNA-ONLY™ Eukaryotic mRNA Isolation Kit, Epicentre). Utilizing a random priming method, each sample was then amplified and transcribed into fluorescent cRNA along the entire length of the transcripts without 3' bias (Arraystar Flash RNA Labeling Kit, Arraystar). The labeled cRNAs were purified using a RNeasy Mini Kit (Qiagen). The concentration and specific activity of the labeled cRNAs (pmol Cy3/μg cRNA) were measured using NanoDrop ND-1000. A sample (1 μg) of each labeled cRNA was fragmented by adding 5 μl of 10× Blocking Agent and 1 μl of 25× Fragmentation Buffer, after which the mixture was heated for 30 min at 60°C. Finally 25 μl of 2× GE Hybridization buffer was added to dilute the labeled cRNA. Aliquots (50 μl) of hybridization solution were dispensed into gasket slides and assembled into the LncRNA expression microarray slide. The slides were incubated for 17 h at 65°C in an Agilent Hybridization Oven. The hybridized arrays were then washed, fixed and scanned using an Agilent DNA Microarray Scanner (part number G2505C).

### Immunohistochemical analysis of KLF5

The immunohistochemical staining protocol was described previously [4, 5]. Briefly, paraffin-embedded tissue sections (4 μm) were immunostained with anti-KLF5 (Proteintech, Chicago, IL, USA). The numbers of KLF5-positive cells were counted randomly in five high-power fields using a microscope (Olympus, Japan).

### Expression and clinical dataset for pancreatic cancer patients

The RNA-sequencing expression profile and related clinical information on pancreatic cancer samples were downloaded from the TCGA data portal [44]. Patients with well-annotated clinical follow up information were retained for further analysis. Ultimately, 177 patients were used in this study.

### Survival analysis

Univariate Cox regression analysis was used to evaluate the association between survival and gene expression in pancreatic cancer patients. The median expression value was used as the cutoff to divide the patients into high and low risk groups. Kaplan-Meier survival curves were constructed for patients in different risk groups, and the statistical significance of differences was assessed using the log-rank test (*P*<0.05). The tumor-free survival curves were plotted using the same strategy used for the Kaplan-Meier survival curves.

### MicroRNA-mRNA interaction network

Important microRNAs regulated by KLF5 were identified as described in the Supplementary Material of reference 25. MicroRNA-mRNA interactions were downloaded from the experimentally verified miRTarBase (24304892) database. Among these interactions, 12 microRNA-mRNA pairs were associated with the 12 of the 677 differentially expressed microRNAs that were up-regulated by KLF5.

### Functional enrichment analysis

The method using for functional enrichment analysis was described previously [20]. Briefly, a hypergeometric test was used to calculate the significance of the enriched genes with respect to GO terms and KEGG pathways. If the whole genome had a total of *N* genes, of which *K* were involved in the biological term or pathway under investigation, and a total of *M* microRNA target genes, of which *x* were involved in the same term or pathway, then the *P* value for the enrichment of that pathway was calculated as follows:

$$P=1-\sum_{t=0}^{x}\frac{\left(\begin{array}{c} K\\ t \end{array}\right)\left(\begin{array}{c} N-K\\ M-t \end{array}\right)}{\left(\begin{array}{c} N\\ M \end{array}\right)}$$

Pathways for which *P*<0.05 were considered significantly enriched.

### Materials

The following antibodies were used: anti-β-actin (Santa Cruz Biotechnology, Carlsbad, CA, USA), anti-KLF5, anti-E2F1, anti-cyclin D1, anti-Rad51, and anti-p16 (Proteintech, Chicago, IL, USA).

### Cell culture

The PANC-1 and BxPC-3 human pancreatic cancer cell lines (American Type Culture Collection, Manassas, VA, USA) were respectively cultured in DMEM and RPMI 1640 medium supplemented with fetal bovine serum (10%), penicillin (100 U/ml), and streptomycin (100 mg/ml) (Irvine Scientific, Irvine, CA, USA). All cells were maintained at 37°C in humidified air with 5% CO_2._ All other reagents were obtained from HyClone China Ltd., Beijing, China. Testing for mycoplasma contamination was done using a Mycoplasma Stain Assay Kit (Beyotime Institute of Biotechnology, Beijing, China). No cell cultures were contaminated with mycoplasma.

### Western blot

Proteins were extracted and western blotted as previously described [20]. The cells were harvested, washed twice in ice-cold PBS, sonicated in RIPA buffer (Beyotime Institute of Biotechnology, Beijing, China) and homogenized. The debris was removed by centrifugation at 12,000 ×*g* for 10 min at 4°C, after which the protein concentrations were determined using a BCA protein assay according to the manufacturer’s instructions. Samples containing equal amounts of protein (50 μg) were separated by electrophoresis on 10% or 15% polyacrylamide SDS gels (100 V for 1 to 2 hours) and transferred to polyvinylidene difluoride (PVDF) membranes by electroblotting (100 V for 1 h at 4°C). The running time and voltage, as well as the transfer time and voltage, required some optimization depending on the circumstances. The membranes were then blocked by incubation for 2 h in 5% skim milk in TBST buffer (TBS plus 0.1% Tween 20), followed by incubation with the appropriate primary antibody overnight at 4°C with gentle agitation. The membranes were then washed several times and incubated with the appropriate horseradish peroxidase-conjugated secondary antibody (Santa Cruz Biotechnology, Carlsbad, CA, USA) for 1 h at room temperature. The membranes were then washed, and the protein bands were visualized using an enhanced chemiluminescence (ECL) kit followed by exposure to X-ray film. β-Actin was simultaneously used as a loading control.

### Transient transfection

siRNA knockdown of KLF5 was performed as previously described [3]. siKLF5-1, siKLF5-2, siKLF5-3 and negative control siRNA (NC) were obtained from RiboBio (Guangzhou, China). Briefly, BxPC-3 cells were plated onto six-well plates and allowed to adhere overnight. The cells were then transfected for 48 hour with each siRNA using a riboFectTM CP transfection kit (RiboBio) according to manufacturer’s instructions. There sequences of the siRNAs were listed as follows: si-KLF5-1 GGACACTCTTAATGTTTCT; si-KLF5-2 GATGTGAAATGGAGAAGTA; si-KLF5-3 GCACAAAAGTTTATACCAA.

### Plasmid construction and transfection

A pcDNA3.1-based, EGFP-expressing plasmid, designated pEGFP-C1, was used to express full-length KLF5 protein. The KLF5 cDNAs were amplified by reverse transcription polymerase chain reaction (RT-PCR) from RNA extracts prepared from cells using TRIzol reagent (Invitrogen). The coding sequence of KLF5 was obtained from KLF5 cDNAs using PCR. Plasmid pEGFP-KLF5 was constructed by inserting the KLF5-coding sequence, which contained 1374 nucleotides (nt) and encoded 457 amino acids (aa), into the EcoR I-Xho I site at the 3’ end of the EGFP-coding sequence in pEGFP-C1. The PCR product were purified using 1% agarose gel electrophoresis and a GeneJET Gel Extraction Kit (Thermo Fisher). The purified PCR products were digested with EcoR I and Xho I (TaKaRa), and ligated to pEGFP-C1 digested with the same restriction enzymes. The plasmids were then confirmed by sequencing (Genewiz). The primers used in these constructions are listed in Table 2 (Supplementary Material). For plasmid transfection, PANC-1 cells plated in 6-well plates were transfected using 3 μg of plasmid with Lipofectamine 2000 (Invitrogen). Mock vector was used as a negative control. After transfection for 48 h, the cells were collected for subsequent experimentation. The efficiency of KLF5-EGFP overexpression was evaluated by western blotting.

**Table 2:** Primers used for plasmid construction.

pf:	CGTCTAGAATTCGCTACAAGGGTGCTGAG
pr:	TATCGACTCGAGTCAGTTCTGGTGCCTCT

### Cell cycle analysis

The method used for DNA cell cycle analysis was described previously [45]. Briefly, cells were collected by trypsinization, washed twice with ice-cold PBS, and fixed for at least 4 h in 2 ml of ice-cold 70% ethanol/30% PBS at 4°C. The ethanol was subsequently removed after centrifugation, and about 1×10^6^ cells were re-suspended in 800 μl of PBS, 100 μl of ribonuclease A (500 μg/ml PBS) and 100 μl of propidium iodide (500 μg/ml PBS) at room temperature in the dark for 30 min. Flow cytometric analysis was then performed using a FACScan (Becton Dickinson) to determine the percentage of cells at different phases of the cell cycle.

### Statistical analysis

Results are expressed as means ± standard deviations (SD). The significance of differences between histopathologic scores was assessed using the Kruskal-Wallis test. Continuous data were analyzed using ANOVA and the Student-Newman-Keuls test. The hypergeometric test was used to identify significantly enriched terms and pathways. Differences were considered statistically significant when *P*<0.05. All statistical analyses were performed using the R 3.1.3 framework.

## Supplementary Material

Supplementary Material

Supplementary Figures

## References

[1] Siegel RL, Miller KD, Jemal A. Cancer statistics, 2018. CA Cancer J Clin. 2018; 68:7–30. 10.3322/caac.21442 29313949

[2] Kamisawa T, Wood LD, Itoi T, Takaori K. Pancreatic cancer. Lancet. 2016; 388:73–85. 10.1016/S0140-6736(16)00141-0 26830752

[3] Liu H, Li L, Chen H, Kong R, Pan S, Hu J, Wang Y, Li Y, Sun B. Silencing IGFBP-2 decreases pancreatic cancer metastasis and enhances chemotherapeutic sensitivity. Oncotarget. 2017; 8:61674–86. 10.18632/oncotarget.18669 28977895 PMC5617455

[4] Kong R, Sun B, Jiang H, Pan S, Chen H, Wang S, Krissansen GW, Sun X. Downregulation of nuclear factor-kappaB p65 subunit by small interfering RNA synergizes with gemcitabine to inhibit the growth of pancreatic cancer. Cancer Lett. 2010; 291:90–98. 10.1016/j.canlet.2009.10.001 19880242

[5] Li L, Chen H, Gao Y, Wang YW, Zhang GQ, Pan SH, Ji L, Kong R, Wang G, Jia YH, Bai XW, Sun B. Long Noncoding RNA MALAT1 Promotes Aggressive Pancreatic Cancer Proliferation and Metastasis via the Stimulation of Autophagy. Mol Cancer Ther. 2016; 15:2232–43. 10.1158/1535-7163.MCT-16-0008 27371730

[6] Sogawa K, Imataka H, Yamasaki Y, Kusume H, Abe H, Fujii-Kuriyama Y. cDNA cloning and transcriptional properties of a novel GC box-binding protein, BTEB2. Nucleic Acids Res. 1993; 21:1527–32. 10.1093/nar/21.7.1527 8479902 PMC309358

[7] Dong JT, Chen C. Essential role of KLF5 transcription factor in cell proliferation and differentiation and its implications for human diseases. Cell Mol Life Sci. 2009; 66:2691–706. 10.1007/s00018-009-0045-z 19448973 PMC11115749

[8] Aizawa K, Suzuki T, Kada N, Ishihara A, Kawai-Kowase K, Matsumura T, Sasaki K, Munemasa Y, Manabe I, Kurabayashi M, Collins T, Nagai R. Regulation of platelet-derived growth factor-A chain by Krüppel-like factor 5: new pathway of cooperative activation with nuclear factor-kappaB. J Biol Chem. 2004; 279:70–76. 10.1074/jbc.M306621200 14573617

[9] Bateman NW, Tan D, Pestell RG, Black JD, Black AR. Intestinal tumor progression is associated with altered function of KLF5. J Biol Chem. 2004; 279:12093–101. 10.1074/jbc.M311532200 14726538

[10] Suzuki T, Sawaki D, Aizawa K, Munemasa Y, Matsumura T, Ishida J, Nagai R. Kruppel-like factor 5 shows proliferation-specific roles in vascular remodeling, direct stimulation of cell growth, and inhibition of apoptosis. J Biol Chem. 2009; 284:9549–57. 10.1074/jbc.M806230200 19189969 PMC2666607

[11] Zhu N, Gu L, Findley HW, Chen C, Dong JT, Yang L, Zhou M. KLF5 Interacts with p53 in regulating survivin expression in acute lymphoblastic leukemia. J Biol Chem. 2006; 281:14711–18. 10.1074/jbc.M513810200 16595680

[12] He M, Han M, Zheng B, Shu YN, Wen JK. Angiotensin II stimulates KLF5 phosphorylation and its interaction with c-Jun leading to suppression of p21 expression in vascular smooth muscle cells. J Biochem. 2009; 146:683–91. 10.1093/jb/mvp115 19628677

[13] Li X, Liu X, Xu Y, Liu J, Xie M, Ni W, Chen S. KLF5 promotes hypoxia-induced survival and inhibits apoptosis in non-small cell lung cancer cells via HIF-1α. Int J Oncol. 2014; 45:1507–14. 10.3892/ijo.2014.2544 25051115

[14] Jia L, Zhou Z, Liang H, Wu J, Shi P, Li F, Wang Z, Wang C, Chen W, Zhang H, Wang Y, Liu R, Feng J, Chen C. KLF5 promotes breast cancer proliferation, migration and invasion in part by upregulating the transcription of TNFAIP2. Oncogene. 2016; 35:2040–51. 10.1038/onc.2015.263 26189798

[15] Zheng HQ, Zhou Z, Huang J, Chaudhury L, Dong JT, Chen C. Krüppel-like factor 5 promotes breast cell proliferation partially through upregulating the transcription of fibroblast growth factor binding protein 1. Oncogene. 2009; 28:3702–13. 10.1038/onc.2009.235 19668233

[16] Xia H, Wang C, Chen W, Zhang H, Chaudhury L, Zhou Z, Liu R, Chen C. Kruppel-like factor 5 transcription factor promotes microsomal prostaglandin E2 synthase 1 gene transcription in breast cancer. J Biol Chem. 2013; 288:26731–40. 10.1074/jbc.M113.483958 23913682 PMC3772219

[17] He P, Yang JW, Yang VW, Bialkowska AB. Kruppel-like Factor 5, Increased in Pancreatic Ductal Adenocarcinoma, Promotes Proliferation, Acinar-to-Ductal Metaplasia, Pancreatic Intraepithelial Neoplasia, and Tumor Growth in Mice. Gastroenterology. 2018; 154:1494–1508.e13. 10.1053/j.gastro.2017.12.005 29248441 PMC5880723

[18] Filipowicz W, Bhattacharyya SN, Sonenberg N. Mechanisms of post-transcriptional regulation by microRNAs: are the answers in sight? Nat Rev Genet. 2008; 9:102–14. 10.1038/nrg2290 18197166

[19] Bartel DP. MicroRNAs: genomics, biogenesis, mechanism, and function. Cell. 2004; 116:281–97. 10.1016/S0092-8674(04)00045-5 14744438

[20] Li Y, Wang Y, Kong R, Xue D, Pan S, Chen H, Sun B. Dihydroartemisinin suppresses pancreatic cancer cells via a microRNA-mRNA regulatory network. Oncotarget. 2016; 7:62460–73. 10.18632/oncotarget.11517 27613829 PMC5308739

[21] Lewis BP, Burge CB, Bartel DP. Conserved seed pairing, often flanked by adenosines, indicates that thousands of human genes are microRNA targets. Cell. 2005; 120:15–20. 10.1016/j.cell.2004.12.035 15652477

[22] Gregory RI, Shiekhattar R. MicroRNA biogenesis and cancer. Cancer Res. 2005; 65:3509–12. 10.1158/0008-5472.CAN-05-0298 15867338

[23] Davis-Dusenbery BN, Hata A. MicroRNA in Cancer: The Involvement of Aberrant MicroRNA Biogenesis Regulatory Pathways. Genes Cancer. 2010; 1:1100–14. 10.1177/1947601910396213 21533017 PMC3083114

[24] Shen Y, Pan Y, Xu L, Chen L, Liu L, Chen H, Chen Z, Meng Z. Identifying microRNA-mRNA regulatory network in gemcitabine-resistant cells derived from human pancreatic cancer cells. Tumour Biol. 2015; 36:4525–34. 10.1007/s13277-015-3097-8 25722110

[25] Zhang B, Zhang Z, Xia S, Xing C, Ci X, Li X, Zhao R, Tian S, Ma G, Zhu Z, Fu L, Dong JT. KLF5 activates microRNA 200 transcription to maintain epithelial characteristics and prevent induced epithelial-mesenchymal transition in epithelial cells. Mol Cell Biol. 2013; 33:4919–35. 10.1128/MCB.00787-13 24126055 PMC3889554

[26] Diaferia GR, Balestrieri C, Prosperini E, Nicoli P, Spaggiari P, Zerbi A, Natoli G. Dissection of transcriptional and cis-regulatory control of differentiation in human pancreatic cancer. EMBO J. 2016; 35:595–617. 10.15252/embj.201592404 26769127 PMC4801945

[27] Gillen S, Schuster T, Meyer Zum Büschenfelde C, Friess H, Kleeff J. Preoperative/neoadjuvant therapy in pancreatic cancer: a systematic review and meta-analysis of response and resection percentages. PLoS Med. 2010; 7:e1000267. 10.1371/journal.pmed.1000267 20422030 PMC2857873

[28] Jia L, Shi Y, Wen Y, Li W, Feng J, Chen C. The roles of TNFAIP2 in cancers and infectious diseases. J Cell Mol Med. . 2018; 22:5188–95. 10.1111/jcmm.13822 30145807 PMC6201362

[29] Yang Y, Tetreault MP, Yermolina YA, Goldstein BG, Katz JP. Krüppel-like factor 5 controls keratinocyte migration via the integrin-linked kinase. J Biol Chem. 2008; 283:18812–20. 10.1074/jbc.M801384200 18450752 PMC2441565

[30] Gao Y, Ding Y, Chen H, Zhou J. Targeting Kruppel-like factor 5 (KLF5) for cancer therapy. Curr Top Med Chem. 2015; 15:699–713. 10.2174/1568026615666150302105052 25732792

[31] Nandan MO, Yoon HS, Zhao W, Ouko LA, Chanchevalap S, Yang VW. Krüppel-like factor 5 mediates the transforming activity of oncogenic H-Ras. Oncogene. 2004; 23:3404–13. 10.1038/sj.onc.1207397 15077182 PMC1351030

[32] Taneyhill L, Pennica D. Identification of Wnt responsive genes using a murine mammary epithelial cell line model system. BMC Dev Biol. 2004; 4:6. 10.1186/1471-213X-4-6 15140269 PMC425575

[33] Qie S, Diehl JA. Cyclin D1, cancer progression, and opportunities in cancer treatment. J Mol Med (Berl). 2016; 94:1313–26. 10.1007/s00109-016-1475-3 27695879 PMC5145738

[34] Liu Y, Wen JK, Dong LH, Zheng B, Han M. Krüppel-like factor (KLF) 5 mediates cyclin D1 expression and cell proliferation via interaction with c-Jun in Ang II-induced VSMCs. Acta Pharmacol Sin. 2010; 31:10–18. 10.1038/aps.2009.185 20037604 PMC4002698

[35] Ertosun MG, Hapil FZ, Osman Nidai O. E2F1 transcription factor and its impact on growth factor and cytokine signaling. Cytokine Growth Factor Rev. 2016; 31:17–25. 10.1016/j.cytogfr.2016.02.001 26947516

[36] Attwooll C, Lazzerini Denchi E, Helin K. The E2F family: specific functions and overlapping interests. EMBO J. 2004; 23:4709–16. 10.1038/sj.emboj.7600481 15538380 PMC535093

[37] Julian LM, Blais A. Transcriptional control of stem cell fate by E2Fs and pocket proteins. Front Genet. 2015; 6:161. 10.3389/fgene.2015.00161 25972892 PMC4412126

[38] Dapas B, Farra R, Grassi M, Giansante C, Fiotti N, Uxa L, Rainaldi G, Mercatanti A, Colombatti A, Spessotto P, Lacovich V, Guarnieri G, Grassi G. Role of E2F1-cyclin E1-cyclin E2 circuit in human coronary smooth muscle cell proliferation and therapeutic potential of its downregulation by siRNAs. Mol Med. 2009; 15:297–306. 10.2119/molmed.2009.00030 19603101 PMC2710289

[39] Farra R, Grassi G, Tonon F, Abrami M, Grassi M, Pozzato G, Fiotti N, Forte G, Dapas B. The Role of the Transcription Factor E2F1 in Hepatocellular Carcinoma. Curr Drug Deliv. 2017; 14:272–81. 10.2174/1567201813666160527141742 27109336

[40] Bhattacharya S, Srinivasan K, Abdisalaam S, Su F, Raj P, Dozmorov I, Mishra R, Wakeland EK, Ghose S, Mukherjee S, Asaithamby A. RAD51 interconnects between DNA replication, DNA repair and immunity. Nucleic Acids Res. 2017; 45:4590–605. 10.1093/nar/gkx126 28334891 PMC5416901

[41] Lundin C, Schultz N, Arnaudeau C, Mohindra A, Hansen LT, Helleday T. RAD51 is involved in repair of damage associated with DNA replication in mammalian cells. J Mol Biol. 2003; 328:521–35. 10.1016/S0022-2836(03)00313-9 12706714

[42] Romagosa C, Simonetti S, López-Vicente L, Mazo A, Lleonart ME, Castellvi J, Ramon y Cajal S. p16(Ink4a) overexpression in cancer: a tumor suppressor gene associated with senescence and high-grade tumors. Oncogene. 2011; 30:2087–97. 10.1038/onc.2010.614 21297668

[43] Dickson MA. Molecular pathways: CDK4 inhibitors for cancer therapy. Clin Cancer Res. 2014; 20:3379–83. 10.1158/1078-0432.CCR-13-1551 24795392

[44] Cancer Genome Atlas Research Network. Comprehensive genomic characterization defines human glioblastoma genes and core pathways. Nature. 2008; 455:1061–68. 10.1038/nature07385 18772890 PMC2671642

[45] Wang Y, Zhou Y, Zhou H, Jia G, Liu J, Han B, Cheng Z, Jiang H, Pan S, Sun B. Pristimerin causes G1 arrest, induces apoptosis, and enhances the chemosensitivity to gemcitabine in pancreatic cancer cells. PLoS One. 2012; 7:e43826. 10.1371/journal.pone.0043826 22952775 PMC3429499

