# Supplementary material for: Overexpression of KLF5 is associated with poor survival and G1/S progression in pancreatic cancer
Source: Aging (Albany NY). 2019 Jul 21;11(14):5035–57. doi: 10.18632/aging.102096 (PMC6682527; doi:10.18632/aging.102096)
Supplement: Supplementary Material [file aging-11-102096-s001.pdf]

SUPPLEMENTARY MATERIALS

MicroRNA microarray procedure

MicroRNA microarray and data analysis were performed by KangChen Biotechnology, Shanghai, China. The procedure was previously described by us [20].

CCK-8 assay

Cell viability among the treated cells was determined by using a Cell Counting Kit-8 (CCK-8) kit (Dojindo Laboratories, Kumamoto, Japan) following the instructions outlined by the manufacturer and as previously described by us [45].
