# Supplementary material for: Overexpression of KLF5 is associated with poor survival and G1/S progression in pancreatic cancer
Source: Aging (Albany NY). 2019 Jul 21;11(14):5035–57. doi: 10.18632/aging.102096 (PMC6682527; doi:10.18632/aging.102096)
Supplement: Supplementary Figures [file aging-11-102096-s002.pdf]

SUPPLEMENTARY FIGURES

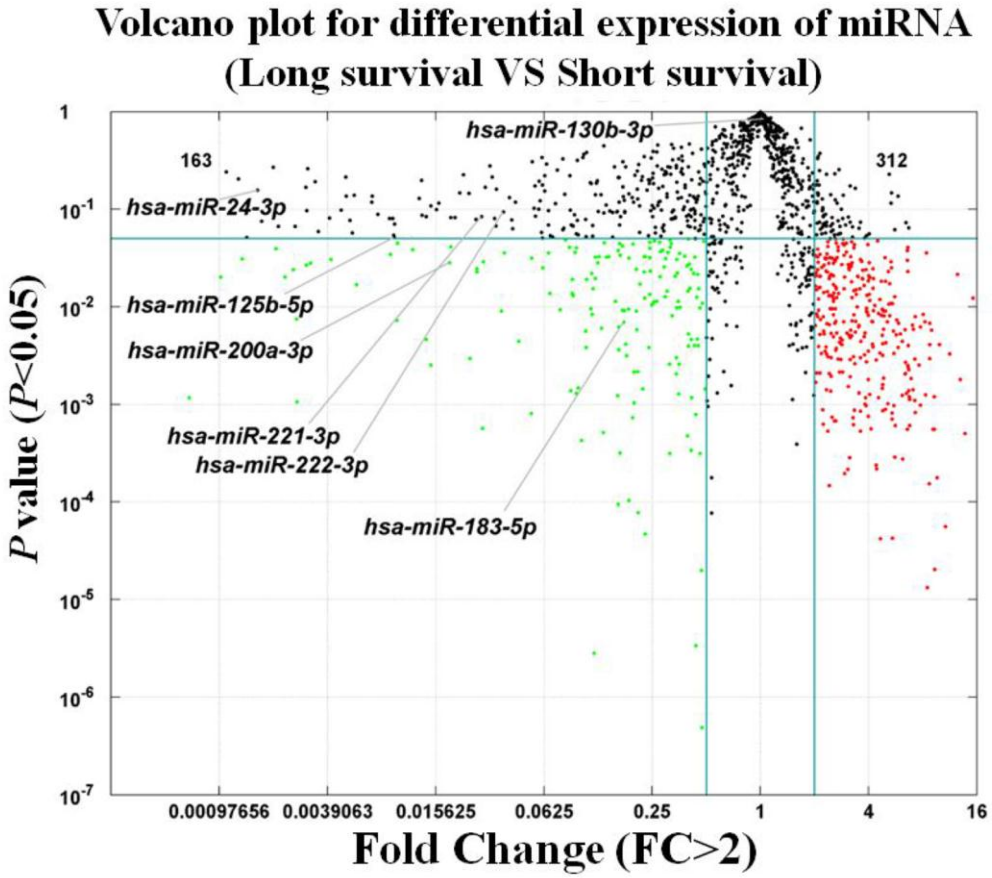

Supplementary Figure 1. Volcano plot illustrating the microRNA gene expression profiles of short- and long-surviving pancreatic cancer patients. Volcano plot showing the differential expression of miRNAs ($P<0.05$, $FC>2$). Expression of miR-24-3p, miR-125b-5p, miR-200a-3p, miR-221-3p, miR-222-3p and miR-183-5p is higher tissue samples from three short-surviving patients than three long-surviving patients. The P values for miR-200a-3p and miR-183-5p are less than 0.05.

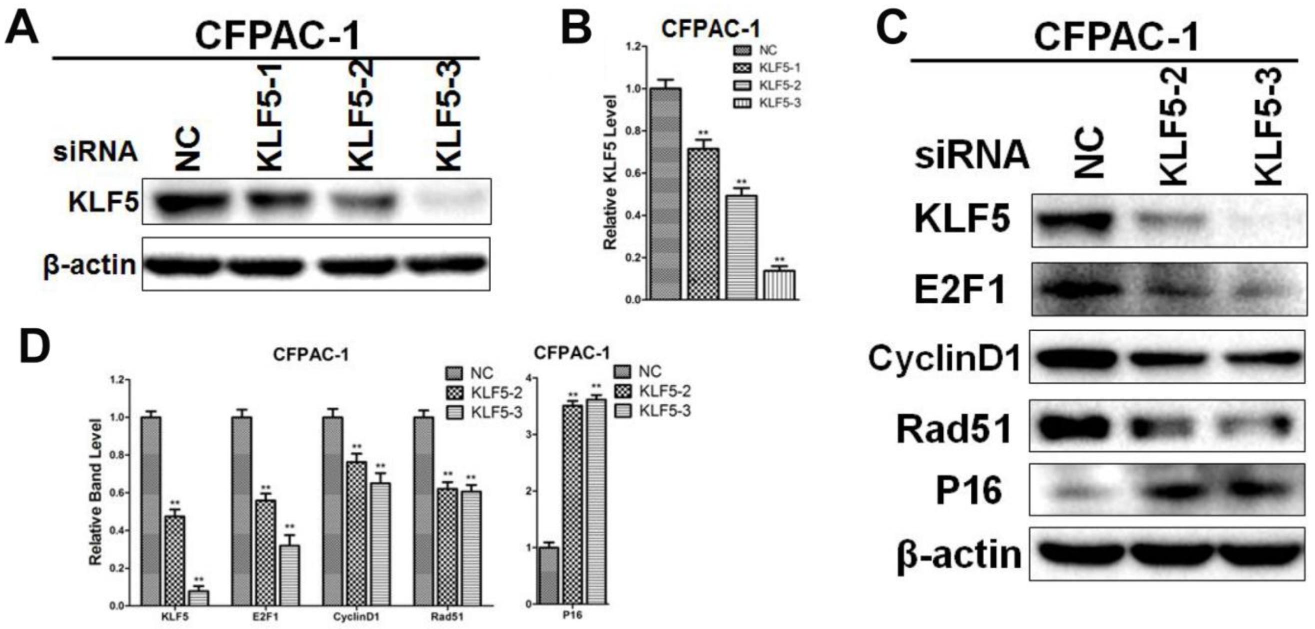

Supplementary Figure 2. Western blot analysis confirming the regulatory relationships between KLF5 and its target genes in CFPAC-1 cells. (**A**) The expression of KLF5 after treatment with KLF5 siRNA in CFPAC-1 cell line. (**B**) The density of each band was measured and normalized to the β-actin band. * *P* <0.05, ** *P* <0.01 vs. the negative control (NC) group. (**C**) CFPAC-1 cells were transfected for 48 h with negative control siRNA, siKLF5-2 or siKLF5-3, after which protein extracts were assayed by western blotting. β-actin was used as a protein loading control. (**D**) The density of each band was measured and normalized to the β-actin band. * *P* <0.05, ** *P* <0.01 vs. the NC group.

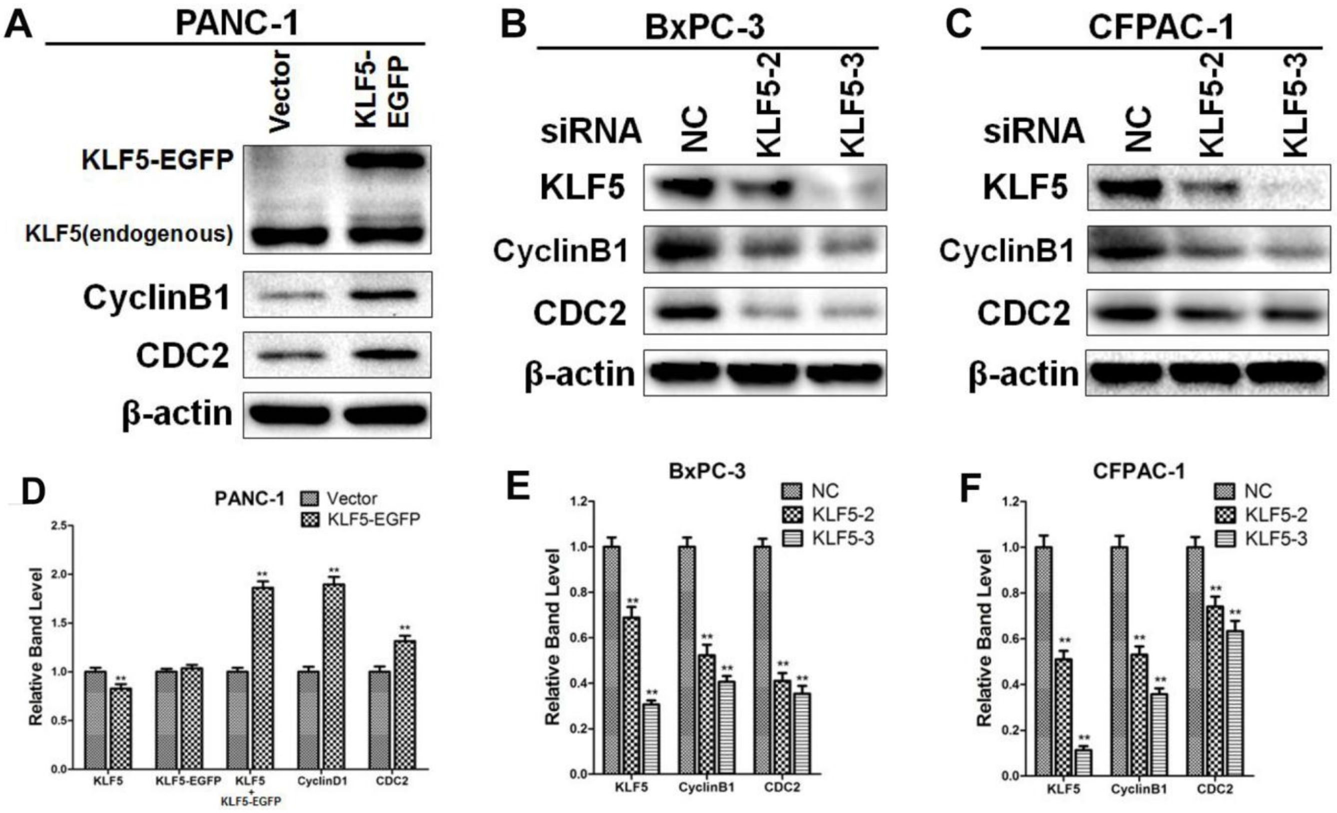

Supplementary Figure 3. Western blot analysis confirming the regulatory relationships between KLF5 and cyclin B1, CDC2. (**A**) PANC-1 cells were transfected for 48 h with empty vector or KLF5-EGFP plasmid, after which protein extracts were assayed by western blotting. β-actin was used as a protein loading control. (**B** and **C**) BxPC-3 (**B**) and CFPAC-1 (**C**) cells were transfected for 48 h with NC siRNA, siKLF5-2 or siKLF5-3, after which protein extracts were assayed by western blotting. β-actin was used as a protein loading control. (**D**–**F**) The density of each band was measured and normalized to the β-actin band. **P* <0.05, ** *P* <0.01 vs. the empty vector group (**D**) or the NC group (**E**, **F**).

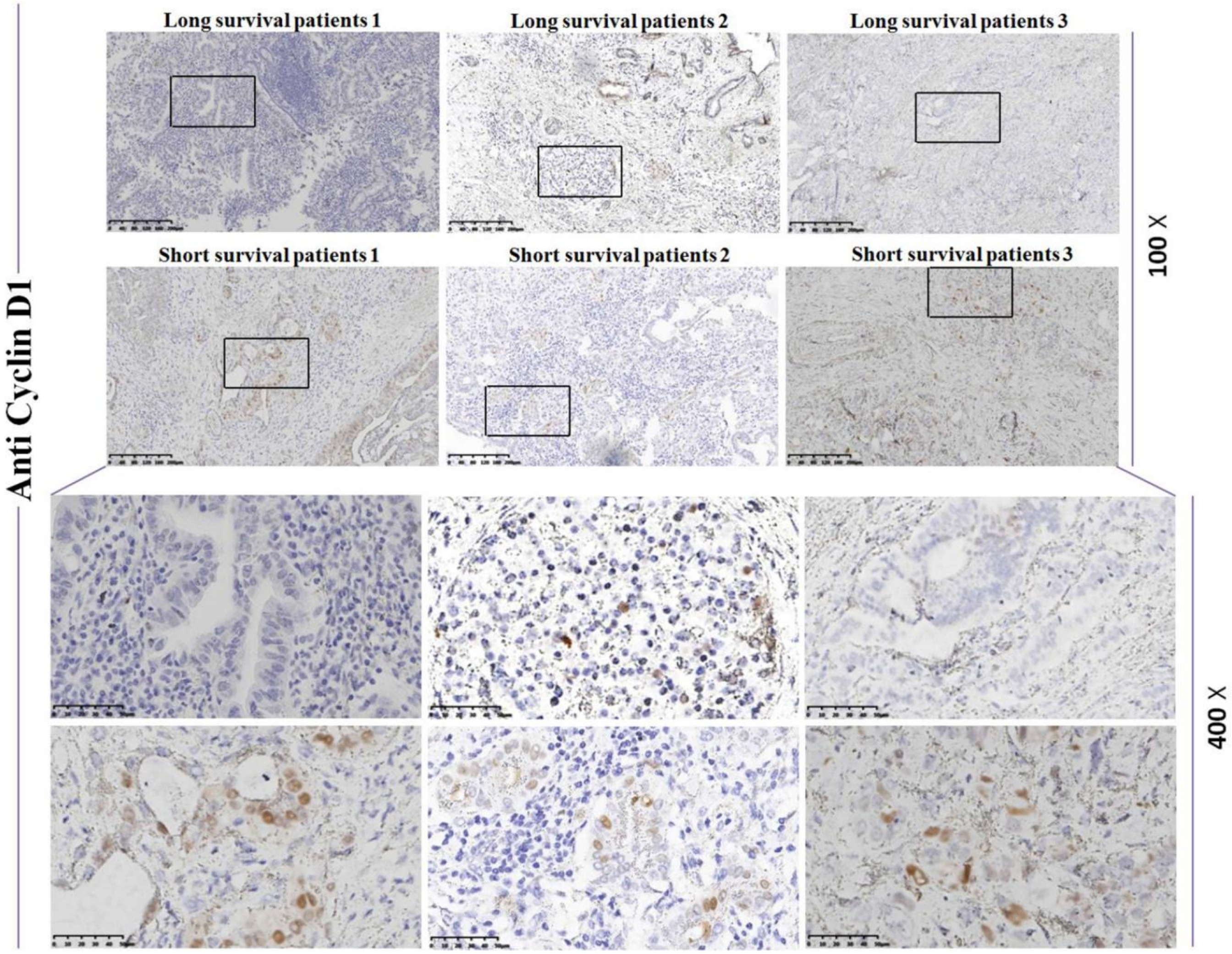

Supplementary Figure 4. Immunohistochemical staining for cyclin D1. Tissue samples from three short-surviving patients showed stronger cyclin D1 staining than did samples from three long-surviving patients.

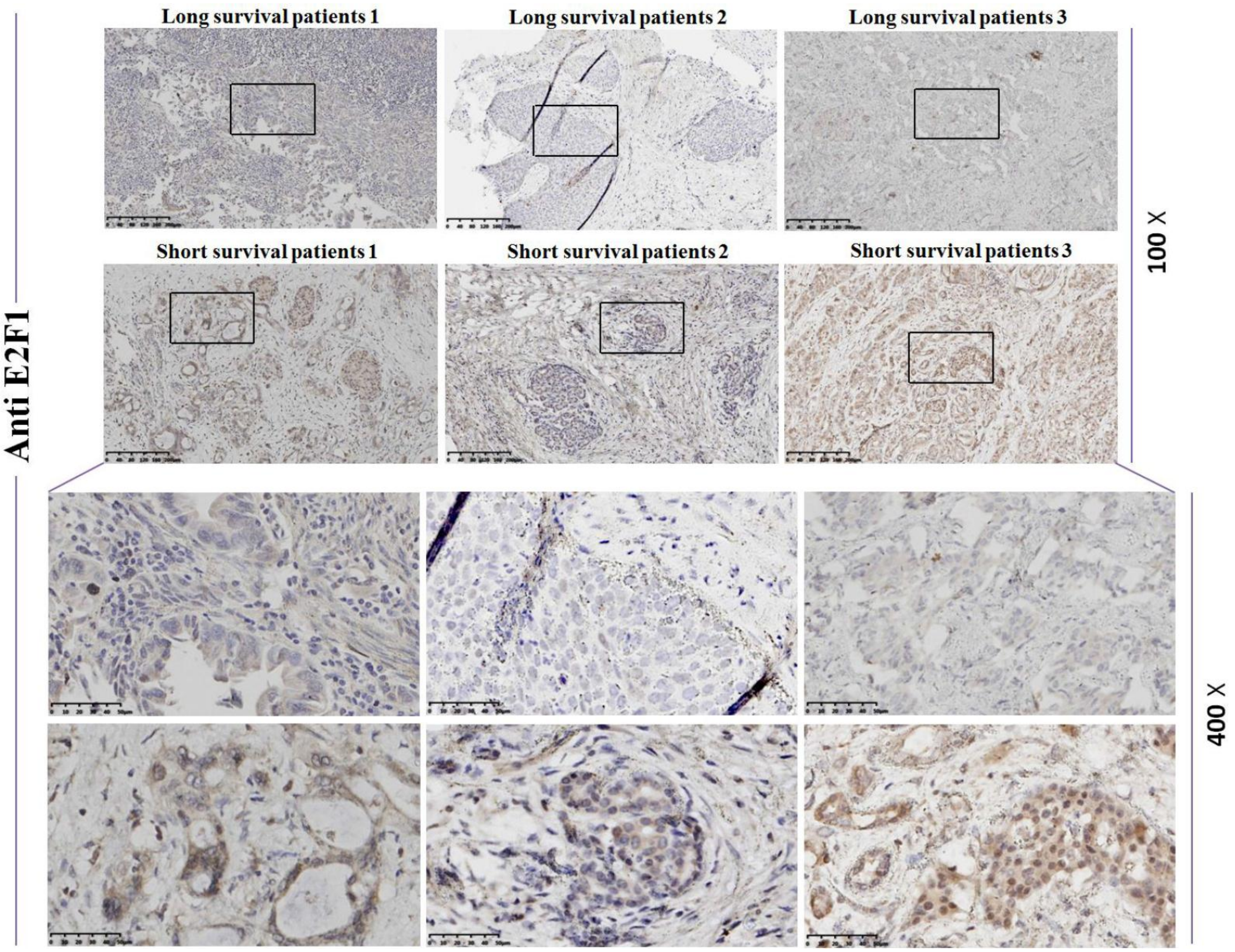

Supplementary Figure 5. Immunohistochemical staining for E2F1. Tissue samples from three short-surviving patients showed stronger E2F1 staining than did samples from three long-surviving patients.

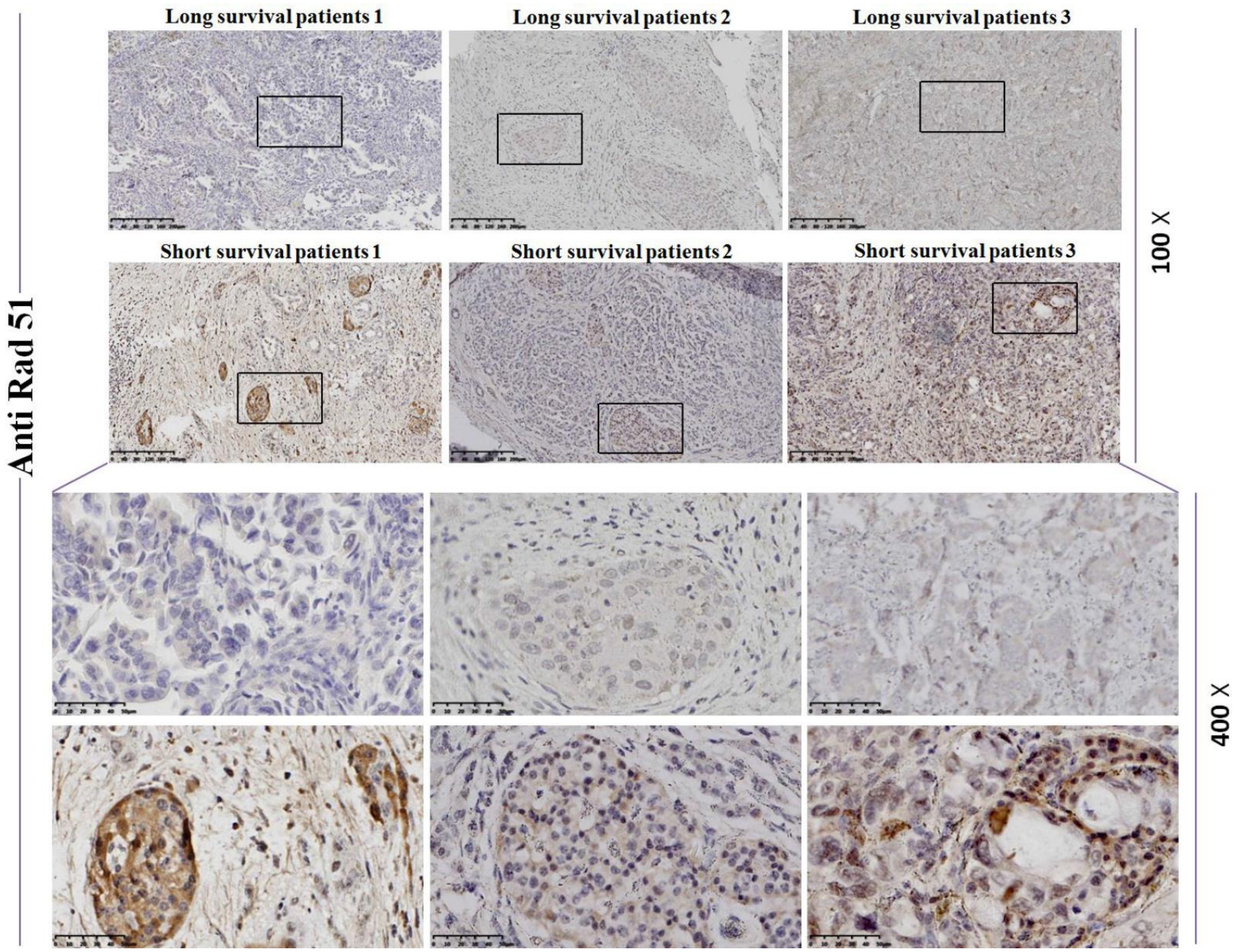

Supplementary Figure 6. Immunohistochemical staining for Rad51. Tissue samples from three short-surviving patients showed stronger Rad51 staining than did samples from three long-surviving patients.

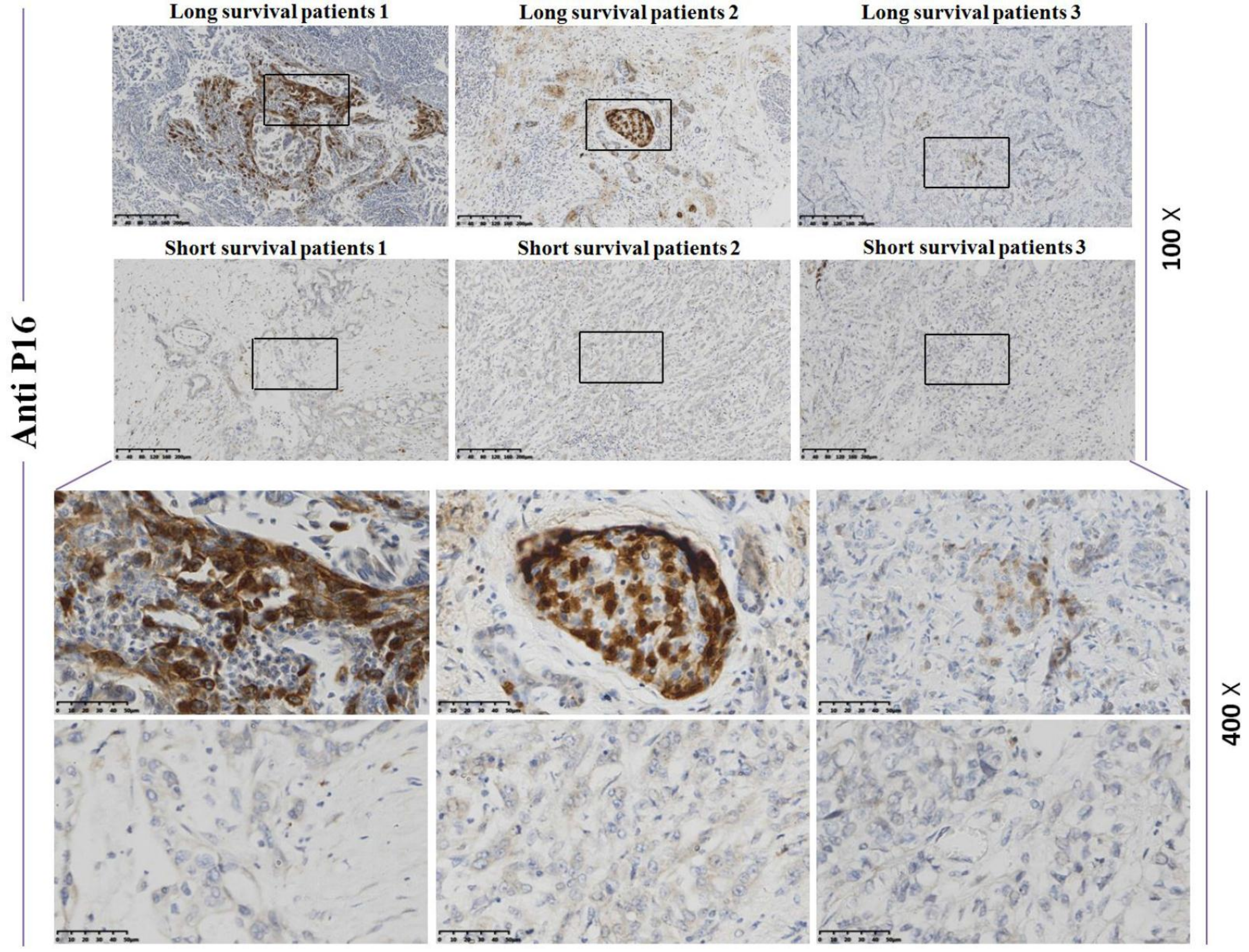

Supplementary Figure 7. Immunohistochemical staining for p16. Tissue samples from three long-surviving patients showed stronger p16 staining than did samples from three short-surviving patients .

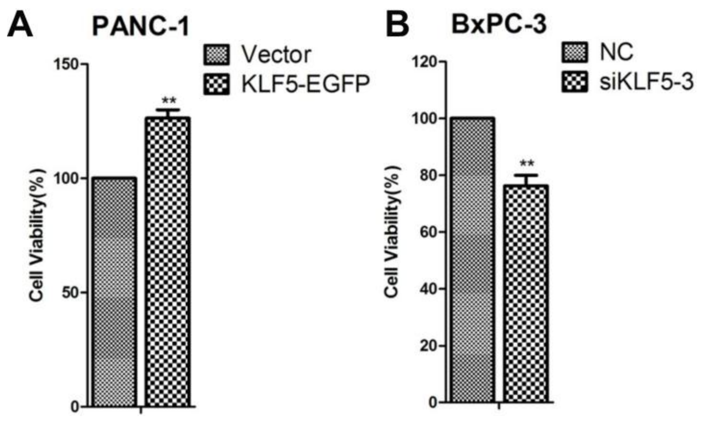

Supplementary Figure 8. Effect of KLF5 on cell viability. (**A**) PANC-1 cells were transfected for 48 h with empty vector or KLF5-EGFP plasmid, after which cell viability was assessed using CCK-8 assays. (**B**) BxPC-3 cells were transfected for 48 h with NC siRNA or siKLF5-3, after which cell viability was assessed using CCK-8 assays.
